# Characterization of the complete mitochondrial genome of the Pamir yak (*Bos grunniens*)

**DOI:** 10.1080/23802359.2019.1667904

**Published:** 2019-09-23

**Authors:** Xian Guo, Xiaoyun Wu, Min Chu, Pengjia Bao, Lin Xiong, Chunnian Liang, Xuezhi Ding, Jie Pei, Ping Yan

**Affiliations:** Key Laboratory of Yak Breeding Engineering of Gansu Province, Lanzhou Institute of Husbandry and Pharmaceutical Sciences, Chinese Academy of Agricultural Sciences, Lanzhou, People’s Republic of China

**Keywords:** Illumina sequencing, iterative mapping, mitogenome, phylogeny, yak

## Abstract

Pamir yak (*Bos grunniens*) is a yak breed from Pamir Plateau with strong adaptation to the high-elevation, cold and anoxic environments. Its complete mitochondrial genome was determined to be 16,323 bp long with a moderately asymmetric nucleotide composition, and harbors the typical set of 37 mitochondrial genes and the noncoding control region. The PCGs start with the typical ATA or ATG codons, and end with TAA, TAG or the incomplete termination codon T. Phylogenetic analysis suggests that Pamir yak is most closely related to six other yak breeds (i.e. Datong, Pali, polled, Qilian, Seron and Sunan yaks).

Domestic yaks (*Bos grunniens*) are mainly distributed in the Qinghai-Tibetan Plateau and adjacent regions, and are of great importance to local communities (Qiu et al. 2012). To date, many local breeds have been developed across its native distribution range (Chu et al. 2016; Guo et al. 2016; Wu, Chu, et al. 2016; Wu, Ding, et al. 2016; Fu et al. 2019; Guo, Bao, et al. 2019; Guo, Wu, et al. 2019; Wu et al. 2019; Zhang et al. 2019; Zhou, Wu, Ding, et al. 2019; Zhou, Wu, Liang, et al. 2019). Among them, Pamir yak is a local breed from Pamir Plateau with strong adaptation to the high-elevation, cold and anoxic environments. Here, we present its complete mitochondrial genome (GenBank accession number: MK922356).

A blood sample of Pamir yak were collected from Tashkurgan Tajik Autonomous County, Xinjiang Uygur Autonomous Region, China (37°10′N, 75°27′E). A voucher specimen is held in the Key Laboratory of Yak Breeding Engineering of Gansu Province, Lanzhou Institute of Husbandry and Pharmaceutical Sciences (Lanzhou, Gansu Province, China). The genomic DNA coded as NO.20190424, which was extracted from Pamir yak, is stored at –80 °C (ultra deep-freeze refrigerator) in the sample storage room of our department. Total genomic DNAs were isolated and purified with the QIAamp DNA Blood Mini Kit (Qiagen, CA, USA). Library preparation and high-throughput sequencing with the Illumina HiSeq X^TM^ Ten Sequencing System (Illumina, CA, USA) were carried out by Annoroad Gene Technology (Beijing, China). Totally, 2.93 Gb of raw data were obtained. Mitogenome assembly was done using MITObim v1.9 (Hahn et al. 2013) with a previously published sequence (JQ692071) (Qiu et al. 2012) as the initial reference. The mitogenome was annotated by comparing with those of its congeners, and necessary adjustments were conducted based on the prediction of the MITOS web server (Bernt et al. 2013; Al Arab et al. 2017).

The mitochondrial genome of Pamir yak is 16,323 bp long, and encodes the typical set of 37 animal mitochondrial genes (13 protein-coding genes/PCGs, 22 tRNAs, and 2 rRNAs). Three PCGs are initiated with ATA (*ND2, ND3* & *ND5*), while all the others use ATG as their start codon. As for the stop codons, one PCG (*ND2*) uses TAG, three PCGs (*COX3, ND3* & *ND4*) use the incomplete stop codon T, while all the others are terminated with TAA. The 22 tRNAs range in length from 60 (*tRNA-Ser^AGN^*) to 75 bp (*tRNA-Leu^UUR^*). The two rRNAs are 957 bp (*12S rRNA*) and 1571 bp (*16S rRNA*) long, respectively, and are separated by *tRNA-Val*. Besides, an 893-bp-long control region occurs between *tRNA-Pro* and *tRNA-Phe*.

A neighbor-joining (NJ) phylogenetic tree was reconstructed using the program MEGA7 (Kumar et al. 2016) to investigate its relationship with other yak breeds with sequenced mitochondrial genomes (Figure 1). All 13 PCGs were used for the phylogenetic analysis. Two *Bison* species, i.e. *Bison bison* (GU946976) (Douglas et al. 2011) and *Bison priscus* (KX269111) (Froese et al. 2017), were included as outgroup taxa. The result suggests that Pamir yak is most closely related to six other yak breeds (i.e. Datong, Pali, polled, Qilian, Seron and Sunan yaks).

**Figure 1.**
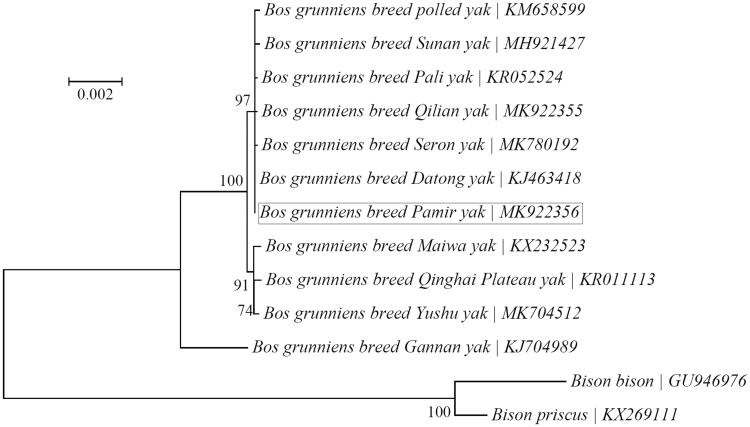
Phylogenetic relationships of 11 yak breeds based on the neighbor-joining (NJ) analysis of the concatenated sequences of 13 mitochondrial protein-coding genes (alignment size: 10,370 bp). The support values next to the nodes are based on 1000 bootstrap replicates. Two *Bison* species were included as outgroup taxa.
